# Proteomics identifies differentially expressed proteins in glioblastoma U87 cells treated with hederagenin

**DOI:** 10.1186/s12953-023-00208-7

**Published:** 2023-04-29

**Authors:** Yesen Zhang, Yi Han, Yuchun Shang, Xiangyu Wang, Jiwei Sun

**Affiliations:** 1 Department of Neurosurgery, The First Affiliated Hospital of Bengbu Medical College, Bengbu, 233004 China; 2 Department of Neurosurgery, The First Affiliated Hospital of Jinan University, Guangzhou, 510630 Guangdong China

**Keywords:** Hederagenin, Proteomics, Tandem mass tags, Glioblastoma, KIF7

## Abstract

**Objective:**

We investigated differentially expressed proteins (DEPs) in human glioblastoma U87 cells after treatment with hederagenin as a therapeutic screening mechanism and provided a theoretical basis for hederagenin in treating glioblastoma.

**Methods:**

The Cell Counting Kit 8 assay was used to analyze the inhibitory effect of hederagenin on the proliferation of U87 cells. Protein was identified by tandem mass tags and LC-MS/MS analysis techniques. Annotation of DEPs, Gene Ontology enrichment and function, and Kyoto Encyclopedia of Genes and Genomes pathways and domains were all examined by bioinformatics. According to the TMT results, hub protein was selected from DEPs for WB verification.

**Results:**

Protein quantitative analysis found 6522 proteins in total. Compared with the control group, 43 DEPs (*P* < 0.05) were involved in the highly enriched signaling pathway in the hederagenin group, among which 20 proteins were upregulated, and 23 proteins were downregulated. These different proteins are mainly involved in the longness regulating pathway–WORM, the hedgehog signaling pathway, *Staphylococcus aureus* infection, complement, coagulation cascades, and mineral absorption. KIF7 and ATAD2B expression were significantly down-regulated and PHEX and TIMM9 expression were significantly upregulated, according to WB analysis, supporting the TMT findings.

**Conclusion:**

Hederagenin inhibition of GBM U87 cells may be related to KIF7, which is mainly involved in the hedgehog signaling pathway. Our findings lay a foundation for additional study of the therapeutic mechanism of hederagenin.

**Supplementary Information:**

The online version contains supplementary material available at 10.1186/s12953-023-00208-7.

## Introduction

The most prevalent and dangerous adult brain tumor is glioblastoma (GBM), distinguished by a high recurrence rate and significant medication resistance. Only 15 months is the median patient survival time [1]. Maximum surgical resection, adjuvant radiation, and temozolomide chemotherapy are currently used to treat GBM [2, 3]. Unfortunately, even with these standardized treatments, GBM often recurs within a few months after surgery. Given the high recurrence rate and low survival of GBM, finding new therapies to overcome the disease is imperative.

Currently, the types and quantities of newly developed anti-tumor drugs are limited across the globe. Still, the number of patients with cancer has gradually increased in the past 10 years, and that number is expected to increase by more than 70% in the next 20 years [4]. Existing cancer therapies have side effects that are destructive or harmful to normal cells in the body. Therefore, anti-tumor drugs with milder and fewer side effects are urgently needed, and an effective strategy to find these anti-tumor drugs is from natural plants [5]. In recent years, pentacyclic triterpenoids have attracted widespread attention because of their intense anti-tumor activity and low cytotoxicity; examples include oleanolic acid, lupinol, and hederagenin [6].

As proteomics technology has rapidly progressed, its aspects, such as protein separation and purification, accurate protein quantification, and other technologies, have gradually matured, making it an important method for studying drug mechanisms. Currently, quantitative methods for mass spectrometry-based proteomics include tandem mass tags (TMTs), Isobaric tags for multiple reaction monitoring, parallel reaction monitoring, and absolute and relative quantification, of which TMT is the most popular [7–9]. TMT technology uses tandem mass spectrometry to identify and measure proteins. It has the advantages of being independent of antibodies, allowing for the simultaneous determination of several samples and targets, and making it simple to establish standard operating procedures. In this study, TMT technology was utilized to identify and examine the proteins with differential expression (DEPs) after hederagenin inhibited GBM cells to determine the anti-glioma mechanism of hederagenin.

## Materials and methods

### Cell culture

GBM U87 cells were bought from the Institute of Basic Medicine’s Cell Center at Peking Union Medical College (China), and they were grown in high-glucose DMEM (Gibco, USA) with 10% fetal bovine serum (Gibco). At 37 degrees Celsius, the cells were incubated with saturated humidity, 5% carbon dioxide, and oxygen. The ratio of the added medium that could be used for a subculture and the total number of cells was approximately 1:3.

### Cell counting kit 8 assay for cell viability assay

100-μl cell suspensions were prepared in 96-well plates, 10,000 cells were added to each well, five compound holes were set, and the plate was precultured in the incubator for 24 h. A medium containing hederagenin (PureChem-Standard Co., Ltd., Chengdu, China) was put on the culture plate in place of the fresh media. Twenty-four hours were spent incubating the plate. Cell Counting Kit 8 (CCK-8) (Biosharp, Hefei, China) solution was added to each well after the medium had been changed. For 1-4 hours, the dish was incubated. A microplate analyzer was used to test the absorbance at 450 nm. Cell viability was calculated as [(OD_HED_ − OD_blank_)/(OD_0_ − OD_blank_)] × 100%, in which OD is optical density and HED is hederagenin. Furthermore, OD_HED_ represents the absorbance of holes with cells, solutions, and drug solutions; OD_blank_ represents the absorbance of holes with culture media and solutions but no cells, and OD_0_ represents the absorbance of holes with cells and solutions but no drug solutions. The quantity of hederagenin digested in U87 cells was also determined using the equation’s results, using the approximate treatment concentration as the 50% inhibitory concentration (IC50).

### Preparation of protein extraction

U87 cells were treated with 40 μg/ml (according to IC50 result) of hederagenin for 24 h on the petri dish and gently cleared twice with low-temperature phosphate-buffered saline; the supernatant was discarded, and an appropriate amount of sodium lauryl sulfonate and Tris-HCl was added for lysis. The cells were rapidly scraped, placed in a boiling water bath for 15 minutes, and then ultrasonically centrifuged at 14000 g for 15 minutes to get the supernatant. It was harvested from the filtrate. Protein quantification was carried out using the bicinchoninic acid technique.

The samples were separated and stored at − 20 °C. A total of 20 μg of protein was added to the 6X loading buffer for each sample, and then for 5 min, the samples were submerged in boiling water. Then, 12% sodium lauryl sulfonate-polyacrylamide gel electrophoresis (constant pressure of 250 V, 40 min) was performed for Coomassie bright blue staining. The filter-aided proteome preparation (FASP) technique was then used to prepare the protein solution [10].

### TMT labeling

Thermo TMT Labeling kit (Thermo Fisher Scientific, Waltham, USA) instructions were followed for labeling 100 g of the peptide from each sample for TMT. Each group’s labeled peptides were combined and graded using an Agilent 1260 Infinity II HPLC system (Agilent Technologies Inc., CA USA). 10 mM of HCOONH_4_ and 5% acetonitrile made up buffer A, whereas 10 mM of HCOONH_4_ and 85% acetonitrile made up buffer B. (pH 10.0). An automated sampler inserted the samples onto the chromatographic column at a flow rate of 1 ml/min after the column had been balanced with liquid A. The liquid phase’s gradient was as follows: Between 0 and 25 minutes, liquid B was at 0%; between 25 and 30 minutes, it had a linear gradient of 0 to 7%; between 30 and 65 minutes, it had a linear ladder of 7 to 40%; between 65 and 70 minutes, it had a linear ladder of 40 to 100%; and between 70 and 85 minutes, it remained at 100%. The components were collected every minute throughout the elution, and the absorbance at 214 nm was measured. Forty elution components were collected in total. After being freeze-dried, the samples were divided into 10 pieces and redissolved in 0.1% formic acid.

### Mass spectrometry analysis

Each sample was separated using an Easy nLC system with a nanoliter flow rate (Thermo Fisher Scientific, USA). Chromatography was used to separate the materials, and a Q Exactive Plus mass spectrometer was used for analysis (Thermo Fisher Scientific, USA). Using a high-resolution mass spectrometer called the Q Exactive Plus, quantitative proteome analysis of TMT was carried out. The original graph files (.raw files) were converted into. mgF files by Q Exactive Plus using Thermo Fisher Scientific’s Proteome Discoverer 2.2 program. The MASCOT2.6 server received the files for database retrieval. The data were then filtered by Proteome Discoverer 2.2 using the standard of a false discovery rate of less than 0.01 after the library file (.dat file) created on the MASCOT server was delivered back to the software. DEPs were indicated by a fold change of more than 1.2 and a *P* value (Student’s t-test) of less than 0.05. Data from protein mass spectrometry were qualitatively analyzed using the Uniprot HomoSapiens _20367 20,200,226 (http://www.uniprot.org). For the mass spectrometry analysis, raw data were employed. Mascot 2.6 and Proteome Discoverer 2.2 were employed for database identification and quantitative analysis.

### Bioinformatics analysis

The target protein set’s alignment sequences with the highest bit scores were retrieved using the Blast2GO Command-Line and the Blast retention findings to produce the Gene Ontology (GO) items (www.geneontology.org). The target protein sequence was mapped to the GO entries obtained throughout the mapping process using the Blast2GO Command-Line. The KEGG Orthology (KO) and Links Annotation, or KOALA, program compared the KEGG genes database using Kyoto Encyclopedia of Genes and Genomes (KEGG) pathway annotations, and KO categorized the target protein sequence. The target protein sequence’s pathway information was automatically derived based on KO categorization. The distribution of each GO classification or KEGG pathway in the target protein and the overall protein was compared by Fisher’s exact test in the GO enrichment annotation or KEGG pathway annotation on the target protein set to assess the level of significance of protein enrichment in a particular GO term or KEGG pathway. Projections were positioned to account for variations in protein analysis using the WOLF PSORT (https://wolfpsort.hgc.jp/) program. We examined the functional domain annotation of various proteins using the InterPro database. The significance level of the enrichment degree in a functional domain was assessed by comparing the distribution of various proteins in the total protein using Fisher’s exact test. The target protein’s quantitative data were adjusted for cluster analysis. A hierarchical clustering heat map was produced using Matplotlib software while classifying samples and protein expression in two dimensions.

### Identification by Western blotting

Choose the hub protein from DEPs for WB confirmation, as the TMT data suggest. In the logarithmic stage, U87 cells were divided into two groups: the control group and the 40 g/mL hederagenin group. After 24 h, U87 cell proteins were collected and eliminated using a RIPA lysis cushion containing 1 mM PMSF from Beijing Applygen Technologies Co., Ltd. (Beijing Applygen Technologies Co., Ltd.). The BCA (Beijing Elabscience Co., Ltd.) technique did not wholly lock down protein. Then, each collection of 20 ng protein samples was deposited onto a PVDF layer using SDS-PAGE and electrophoresis (Millipore, USA). The layer was inhibited with 5% skim milk for 1 hour and short-term, at 4 degrees Celsius, brooded with necessary antibodies. The layer was treated with TBST (Beijing Applygen Technologies Co., Ltd.) several times the next day before being brooded for 1 h at room temperature with optional antibodies.

TBST was used to repeatedly wash the layer before ECL was used to identify the goal groups (Thermo, USA). To normalize protein levels, −actin (Beijing zsbio, Inc.) was used as the internal control. KIF7 is one of the crucial antibodies used in this investigation (Proteintech Group, Inc. USA). Additional antibodies came from Beijing zsbio.

## Results

### Inhibitory effect of hederagenin on U87 cell proliferation

**Fig. 1:**
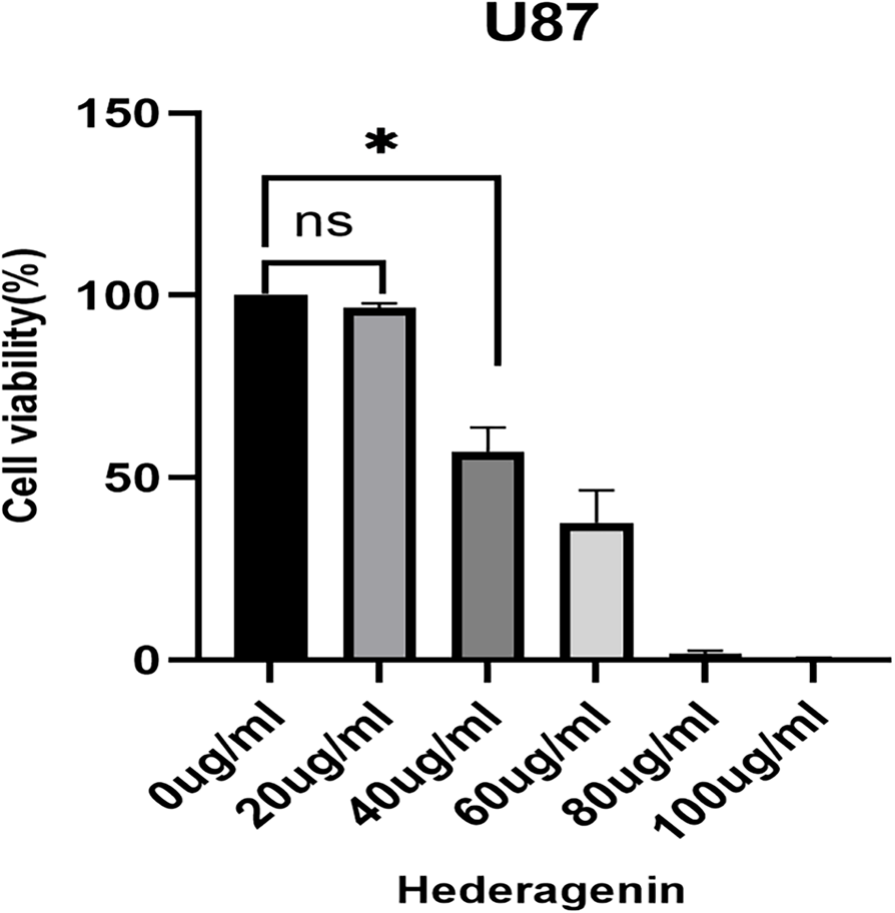
Hederagenin inhibits U87 cell proliferation. ns, no significant difference; * *P* < 0.05

**Fig. 2:**
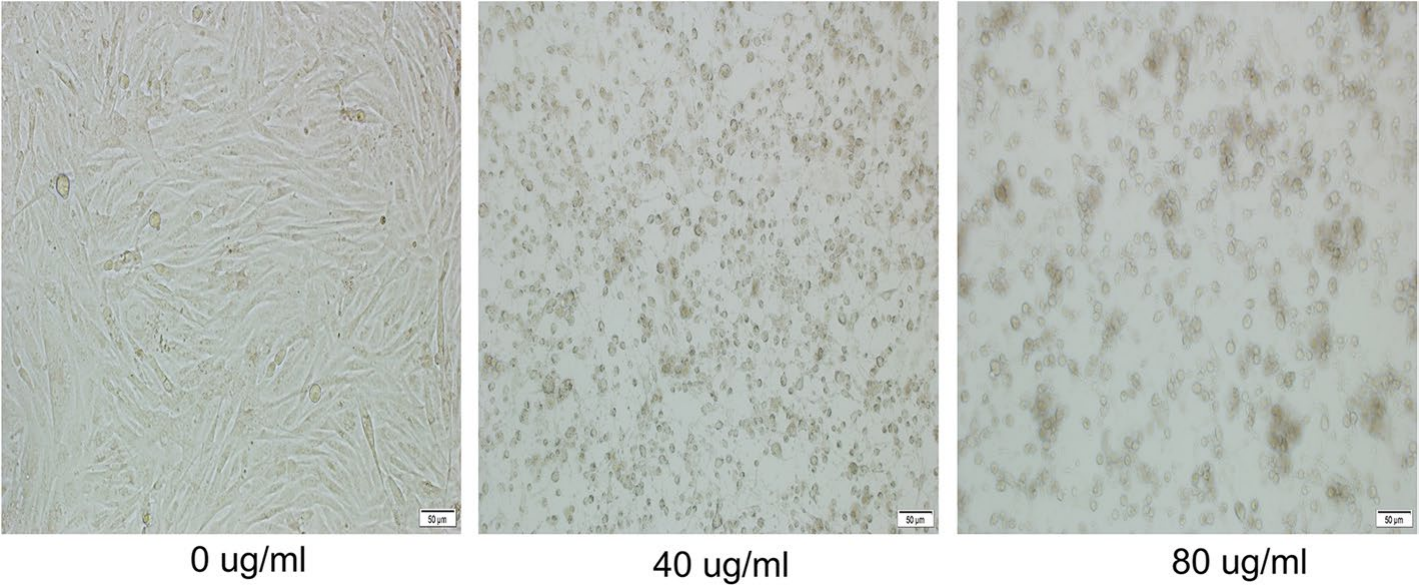
Hederagenin induces apoptosis in U87 cells

Hederagenin inhibited GBM cell proliferation across different treatment concentrations in U87 cells for 24 h. Hederagenin was the treatment group, and no hederagenin was the control group; CCK-8 detected cell proliferation (Fig. 1). Cells treated with 20 μg/ml of hederagenin showed no significant inhibition of the cell proliferation rate (*P* = 0.0549). In contrast, treatment with more than 40 μg/ml significantly inhibited the U87 cell line (*P* < 0.05) (Fig. 2).

### Differential protein expression

**Fig. 3:**
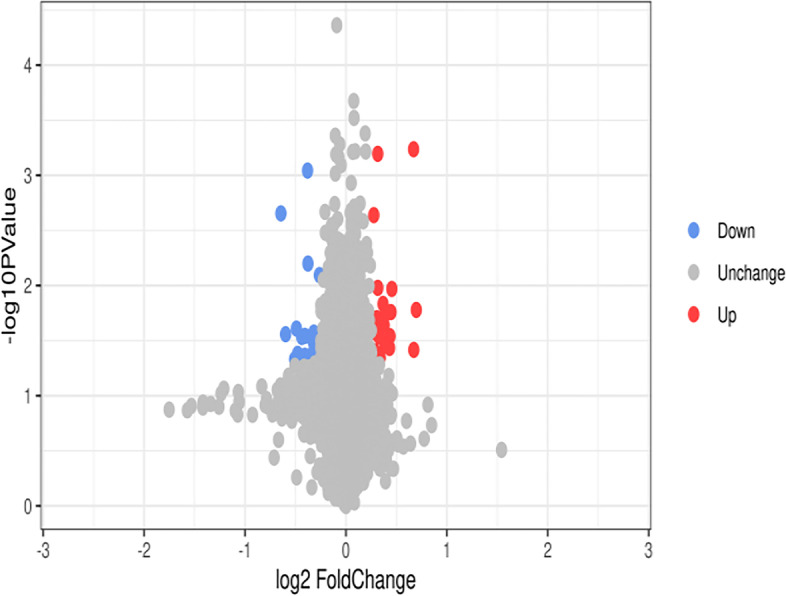
Differential proteins volcano plot. The blue circle on the left represents the downregulated protein number, and the red circle on the right represents the upregulated protein number. The abscissa is the difference multiple; the ordinate is the *P* value

A TMT-labeled quantitative proteomics method was used to analyze the difference in protein expression in glioma cells after inhibition by hederagenin. SDS-PAGE electrophoresis confirmed that the total protein samples had good parallelism, clear protein bands, and no protein degradation (Supplementary Data S1). Forty-three DEPs were identified by database retrieval of the original mass spectrometry data, among which 20 proteins were significantly upregulated and 23 were significantly downregulated (Table 1). Volcanic and heat maps of differential protein expression are shown in Fig. 3 and Supplementary Data S2. The subcellular localization of the differential protein is shown in Supplementary Data S3.

**Table 1:** Differential proteins analysis list

Accession	Gene Name	Description	Regulation	Ratio	*P* value
P78562	PHEX	Phosphate-regulating neutral endopeptidase	UP	1.620795107	0.01664201
P62328	TMSB4X	Thymosin beta-4	UP	1.593601383	0.03837365
O75506	HSBP1	Heat shock factor-binding protein 1	UP	1.591360691	0.000579744
O60220	TIMM8A	Mitochondrial import inner membrane translocase subunit Tim8 A	UP	1.370209403	0.01073901
Q96JA4	MS4A14	Membrane-spanning 4-domains subfamily A member 14	UP	1.362204724	0.01740508
P06703	S100A6	Protein S100-A6	UP	1.35347195	0.02884026
P18859	ATP5PF	ATP synthase-coupling factor 6, mitochondrial	UP	1.349256069	0.03682002
P80297	MT1X	Metallothionein-1X	UP	1.308964987	0.03463186
Q9Y5J7	TIMM9	Mitochondrial import inner membrane translocase subunit Tim9	UP	1.300613497	0.02299285
Q9Y5J9	TIMM8B	Mitochondrial import inner membrane translocase subunit Tim8 B	UP	1.297090352	0.01946411
P01344	IGF2	Insulin-like growth factor II	UP	1.289694656	0.01468609
P62273	RPS29	40S ribosomal protein S29	UP	1.265861027	0.0451047
P04004	VTN	Vitronectin	UP	1.25516723	0.02905154
Q86UE8	TLK2	Serine/threonine-protein kinase tousled-like 2	UP	1.244668911	0.01049691
Q6ZR37	PLEKHG7	Pleckstrin homology domain-containing family G member 7	UP	1.2442948	0.000637691
Q9C005	DPY30	Protein dpy-30 homolog	UP	1.237597911	0.01979912
Q96EX3	WDR34	WD repeat-containing protein 34	UP	1.221399482	0.03971936
P04732	MT1E	Metallothionein-1E	UP	1.216925351	0.02659849
P02649	APOE	Apolipoprotein E	UP	1.210759027	0.002296388
Q6UX53	METTL7B	Methyltransferase-like protein 7B	UP	1.204628949	0.02206929
O60291	MGRN1	E3 ubiquitin-protein ligase MGRN1	DOWN	0.833180568	0.00804476
Q9UBW7	ZMYM2	Zinc finger MYM-type protein 2	DOWN	0.823154057	0.03109416
Q9NUE0	ZDHHC18	Palmitoyltransferase ZDHHC18	DOWN	0.823154057	0.03070765
Q9BSL1	UBAC1	Ubiquitin-associated domain-containing protein 1	DOWN	0.822600243	0.02781847
P54252	ATXN3	Ataxin-3	DOWN	0.822546338	0.02871469
Q567V2	MPV17L2	Mpv17-like protein 2	DOWN	0.814333233	0.04897639
Q6P4Q7	CNNM4	Metal transporter CNNM4	DOWN	0.811594203	0.0382056
O94761	RECQL4	ATP-dependent DNA helicase Q4	DOWN	0.804511278	0.03597904
Q9H4B0	OSGEPL1	Probable tRNA N6-adenosine threonylcarbamoyltransferase, mitochondrial	DOWN	0.801801802	0.02674267
Q53GG5	PDLIM3	PDZ and LIM domain protein 3	DOWN	0.798022176	0.0319602
Q9Y4D1	DAAM1	Disheveled-associated activator of morphogenesis 1	DOWN	0.796945193	0.04132556
Q2M1P5	KIF7	Kinesin-like protein KIF7	DOWN	0.796407186	0.03024119
Q9UGL1	KDM5B	Lysine-specific demethylase 5B	DOWN	0.770138684	0.006316176
Q8TD22	SFXN5	Sideroflexin-5	DOWN	0.768051872	0.000904919
Q99640	PKMYT1	Membrane-associated tyrosine - and threonine-specific cdc2-inhibitory kinase	DOWN	0.754385965	0.0433306
Q6UB99	ANKRD11	Ankyrin repeat domain-containing protein 11	DOWN	0.754385965	0.02863485
Q9H7C9	AAMDC	Mth938 domain-containing protein	DOWN	0.738122827	0.02926776
P02533	KRT14	Keratin, type I cytoskeletal 14	DOWN	0.71743487	0.04167076
P01024	C3	Complement C3	DOWN	0.713796058	0.04877362
Q8NFP7	NUDT10	Diphosphoinositol polyphosphate phosphohydrolase 3-alpha	DOWN	0.711554922	0.0245815
Q5H9J9	TCP11X2	T-complex protein 11 X-linked protein 2	DOWN	0.70261067	0.04676847
P81605	DCD	Dermcidin	DOWN	0.660669803	0.02766569
Q9ULI0	ATAD2B	ATPase family AAA domain-containing protein 2B	DOWN	0.639518994	0.002214818

### GO functional annotation and enrichment analysis

**Fig. 4:**
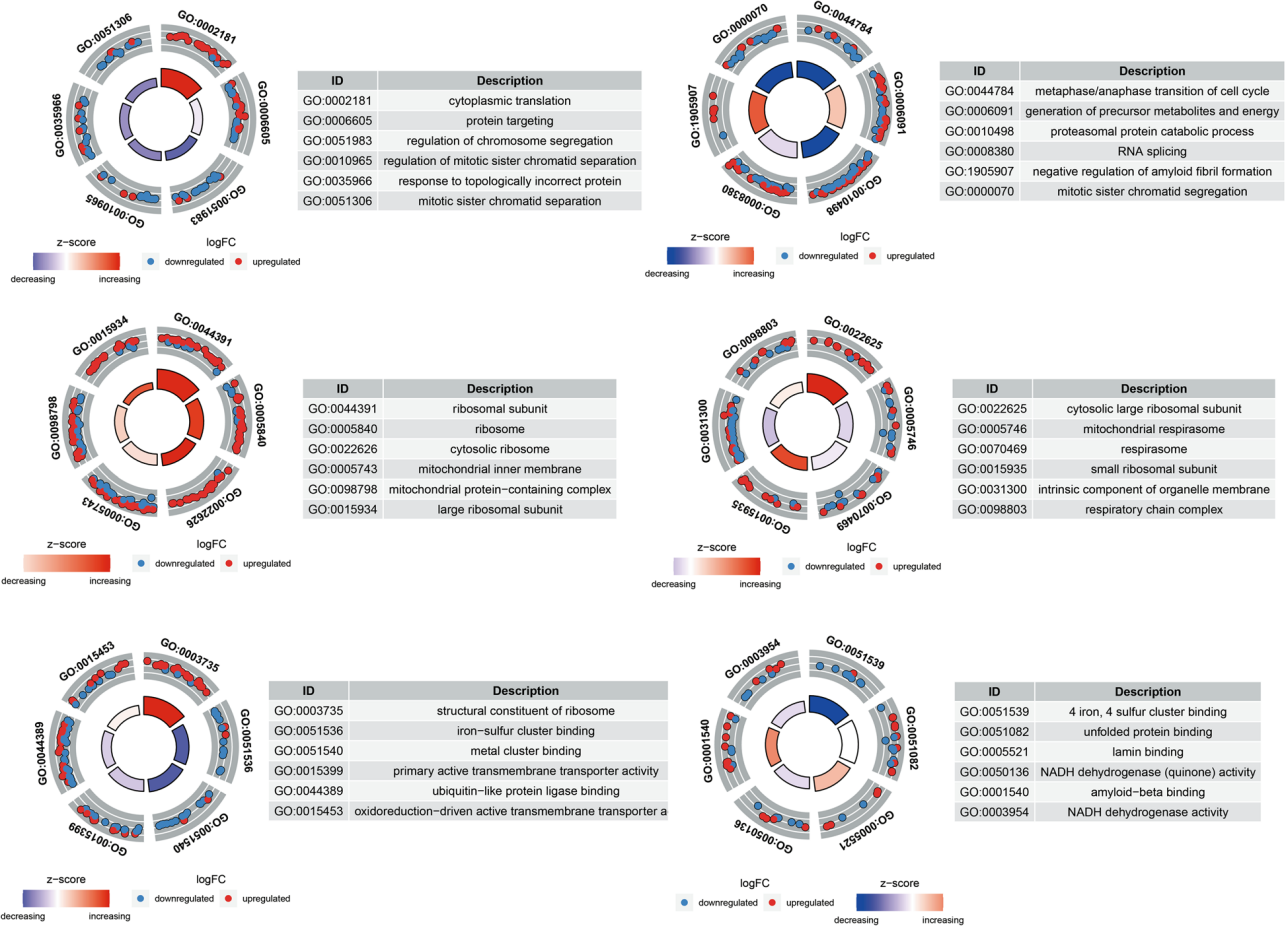
Functional annotation. The properties of differentially expressed proteins (DEPs) in organisms

Figure 4 and Supplementary Data S4 display the results of the GO functional annotation and significant enrichment analyses, respectively. Different proteins were screened and were mostly found to be involved in cellular activities, biological control, the organization of cellular components, or biogenesis. The molecular activity mainly entails binding and catalytic processes. Most of the cellular components consist of cells, organelle parts, and organelles.

### KEGG pathway annotation

**Fig. 5:**
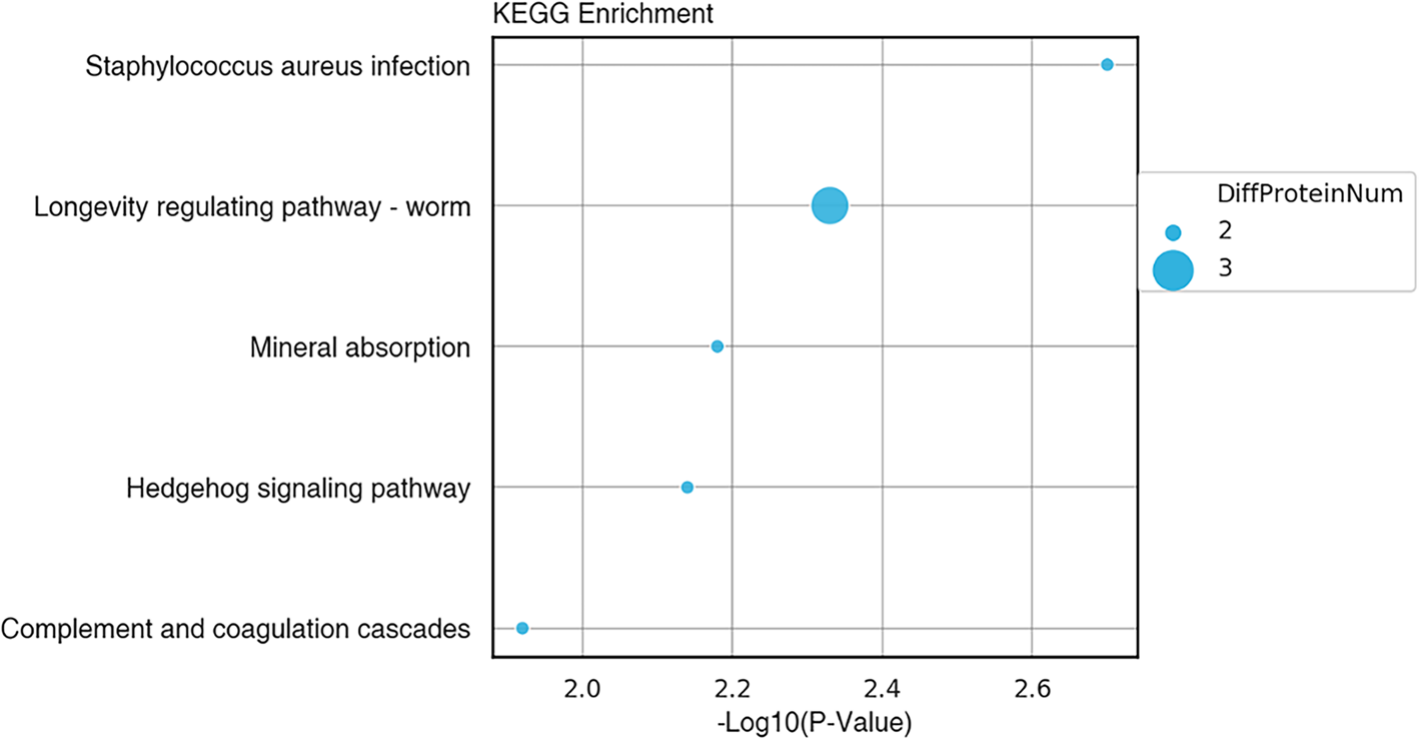
Domain annotations. The top 10 classification results of the most significant enrichment

The KEGG pathway enrichment annotations are shown in Fig. 5. The different KEGG pathway proteins of U87 cells treated with hederagenin and untreated mainly included the longness regulating pathway–WORM, the hedgehog signaling pathway, mineral absorption, *Staphylococcus aureus* infection, and complement and coagulation cascades.

### Domain annotations

The domain annotation results of the hederagenin-treated group versus the untreated group are shown in Supplementary Data S5. Different protein fragments existed mainly in the following proteins: TIM10-like, TIM10-like domain superfamily and mitochondrial import inner membrane translocase submit TPI Tim8/13, metallothionein, metallothionein domain superfamily vertebrate, and heat shock factor binding-1.

### Western blot verification

**Fig. 6:**
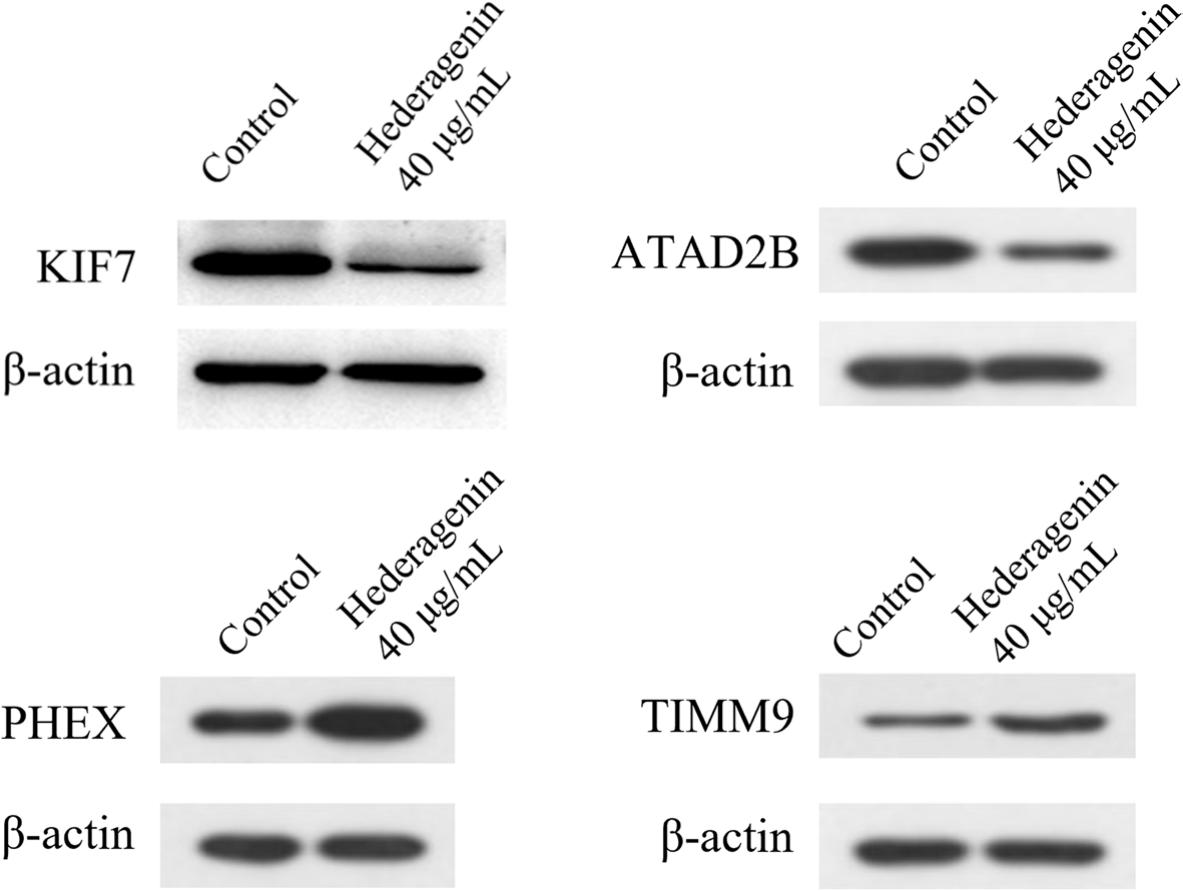
Western Blot Verification of KIF7, ATAD2B, PHEX and TIMM9. The protein expression of KIF7 and ATAD2B were significantly down-regulated when U87 was treated with 40 μg/mL hederagenin, while PHEX and TIMM9 were significantly up-regulated.

KIF7(P06493) was selected from the hub proteins in accordance with the results of the KEGG pathway annotation, and Western blot did not wholly confirm the protein articulation. As demonstrated in Fig. 6, the protein articulation of KIF7 in the hederagenin (40 μg/mL) treatment group was lower than that of the benchmark group. We also tested the expression of ATAD2B, PHEX and TIMM9. ATAD2B was highly expressed in the hederagenin treatment group, while PHEX and TIMM9 expression were lower in hederagenin treatmen group. The results of western blot assays further increased the credibility of the TMT results.

## Discussion

Hederagenin is a pentacyclic triterpenoid compound extracted from the fruit of the soapberry. The main component is soapberry. Hederagenin has anti-inflammatory [11, 12], anti-fungal [13],anti-leishmaniasis [14, 15], anti-neurodegenerative [16], antimicrobial [17], and anti-tumor activities [18, 19]. It has a strong cytotoxic effect on some tumors. Hederagenin increases apoptosis by suppressing the anti-apoptotic protein Bcl-2 in tumor cells, and it primarily stimulates the mitochondrial endogenous apoptosis pathway by activating polyadenosine diphosphate ribose polymerase, caspase-3, caspase-9, and Bax. Hederagenin possesses anti-tumor properties against lung, liver, ovarian, breast, and colon cancers [20–25].

In this study, hederagenin had an inhibitory effect on GBM. Under the light microscope, the number of cells was reduced, the adherent wall was not tight, and some cells were suspended. As the drug concentration increased, inhibition of U87 cell proliferation increased, indicating that hederagenin inhibited GBM cell proliferation in a concentration-dependent manner.

The mechanism of hederagenin inhibition on GBM was examined using quantitative proteomics analysis based on TMT. Twenty proteins showed a substantial upregulation, and 23 showed a significant downregulation among the 43 DEPs. The difference proteins’ GO functional annotation, KEGG pathway annotation, and domain annotation revealed that the differential proteins of hederagenin inhibiting GBM and not inhibiting GBM were mainly concentrated in five pathways; among them, the longevity regulating pathway–WORM and the hedgehog signaling pathway are involved in related inhibitory effects in other tumors [26, 27]. They could also play a role in how hederagenin inhibits the growth of gliomas.

The longevity-regulating pathway–WORM is a stress-induced heat shock reaction. HSBP-1 negatively regulates the life span of cells in the pathway, whereas the life span of cells is favorably regulated by heat shock transcription factor 1 (HSF-1). Furthermore, related studies have shown that HSBP-1 negatively regulates the expression of HSF-1 [28, 29]. Breast and lung cancer exhibit aberrant expression of HSF-1, indicating a direct correlation between the tumor’s level of malignancy and its ability to metastasize. Cantharidin and vitexin promote cell apoptosis by inhibiting this pathway in colorectal cancer cells [26, 30].

Both drosophila and human have a high degree of conservation in the hedgehog signaling pathway. It is involved in developing the central neural tube, the digestive tube, the bronchus, bone, cartilage, the lung, and other organs [31]. Three ligands make up the hedgehog pathway in mammals: the Indian hedgehog, the Desert hedgehog, and the Sonic Hedgehog. Sonic Hedgehog is the most effective of these three and is also the most prevalent in adult tissues [32]. Hedgehog pathway receptors include patched receptors, smoothened receptors, and the transcription factor protein family distributed on the target cell membrane. The main corrector protein is the suppressor of the fused homolog. Its downstream molecules are kinesin family member 7(KIF7) and protein kinase A; among these, the smoothened receptor is an essential regulator in the hedgehog signaling pathway [31]. We found that KIF7 in this pathway was significantly decreased and inhibited cell proliferation.

Jervine reportedly promotes autophagy of tumor cells by blocking the hedgehog signaling pathway, and it has an anti-tumor effect on nasopharyngeal carcinoma [33]. By blocking signaling pathways, including hedgehog and Wnt, lapatinib prevented the development of non-small cell lung cancer cells and lung cancer tumor stem cells [27].

In this research, quantitative proteomics by TMT tagging effectively filtered out the different hederagenin proteins related to its actions in GBM, and the corresponding biological analysis improved understanding of the involvement of these proteins in the inhibitory mechanism of hederagenin in GBM. Western Blot validated the hub protein of KIF7. These findings provide new ideas for research about GBM treatment; however, screening hub proteins is only the first step, and additional verification through subsequent experiments is needed.

## Conclusion

In summary, Through the hedgehog signaling system, hederagenin can reduce the growth of glioma cells and may encourage tumor cell death. These findings lay a foundation for additional study of the therapeutic mechanism of hederagenin.

## Supplementary Information

**Additional file 1: Supplementary Data S1.** SDS-PAGE electrophoresis. **Supplementary Data S2.** Heat maps of differential protein expression. **Supplementary Data S3.** The subcellular localization of the differential protein. **Supplementary Data S4.** Significant enrichment analysis. **Supplementary Data S5.** The domain annotation results of the hederagenin-treated group versus the untreated group.

## Data Availability

Data in this article are available from the corresponding author upon request.
