# Supplementary material for: Proteomics identifies differentially expressed proteins in glioblastoma U87 cells treated with hederagenin
Source: Proteome Sci. 2023 Apr 29;21:7. doi: 10.1186/s12953-023-00208-7 (PMC10148390; doi:10.1186/s12953-023-00208-7)

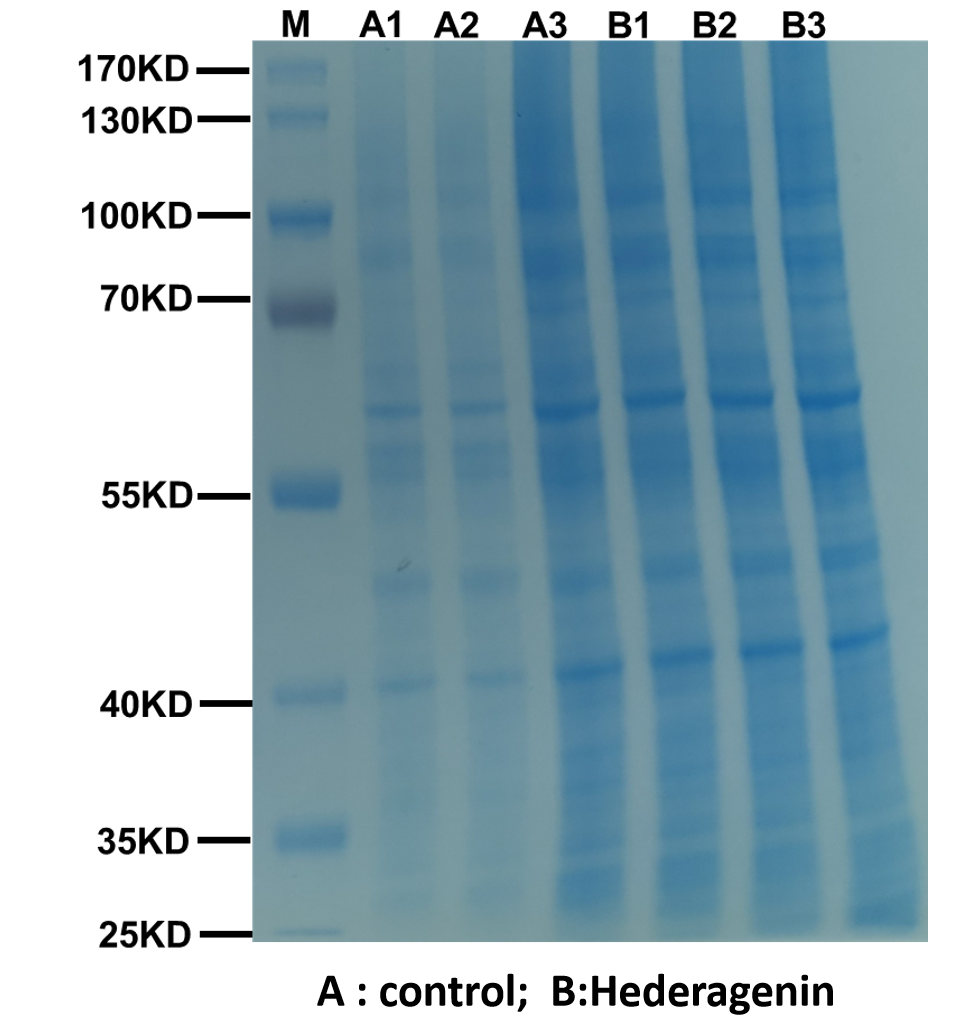


**Supplementary Data S1：**SDS-PAGE electrophoresis.


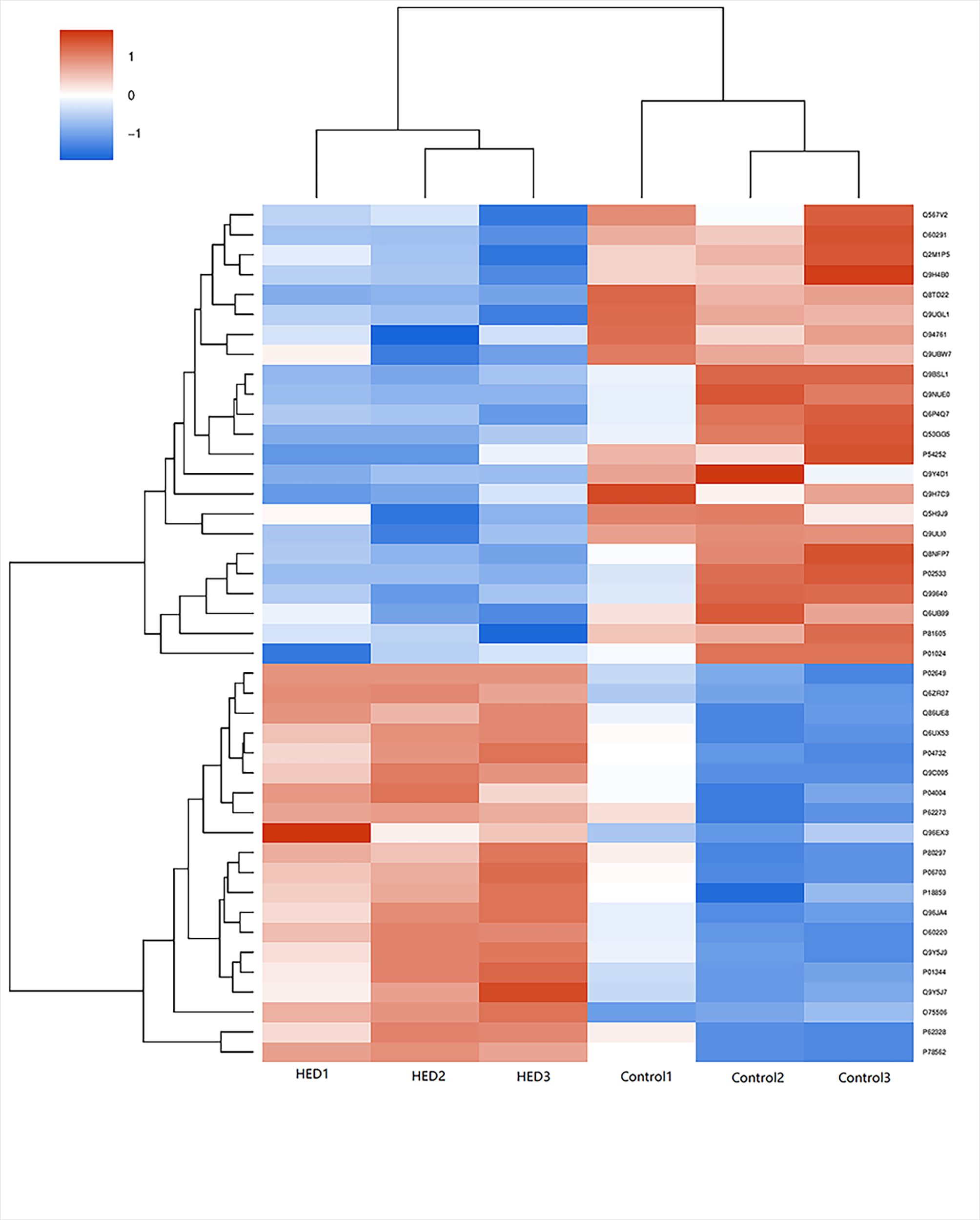


**Supplementary Data S2：**Heat maps of differential protein expression.


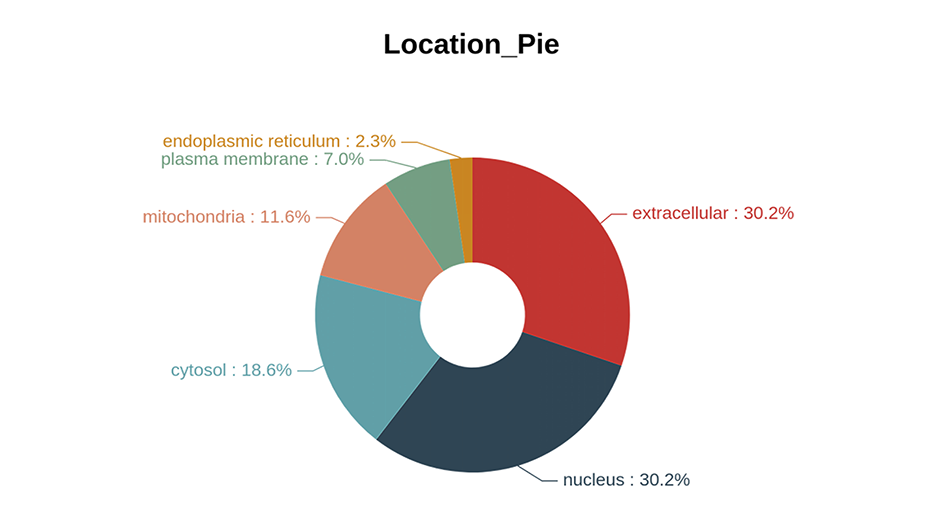


**Supplementary Data S3：**The subcellular localization of the differential protein.


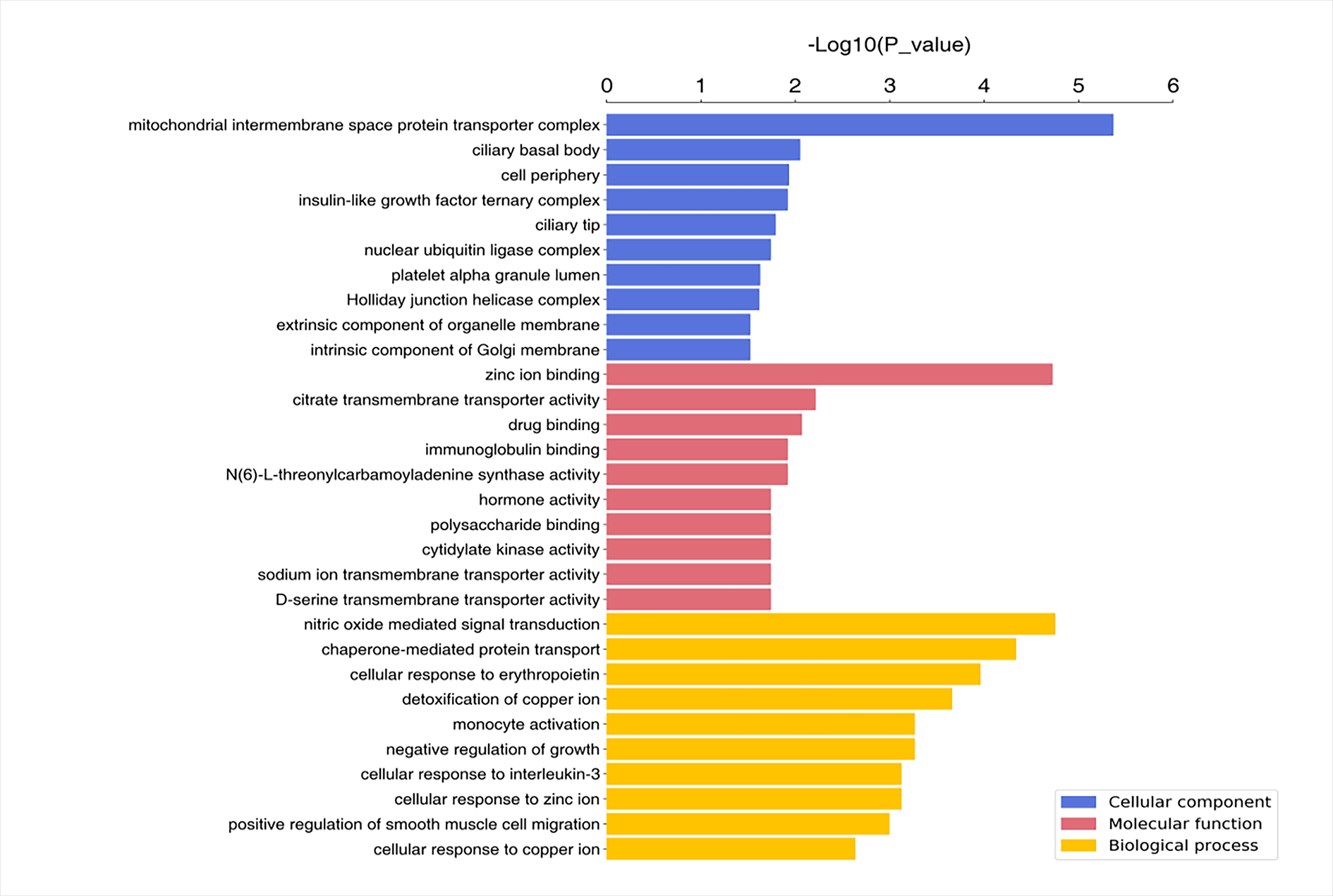


**Supplementary Data S4:** Significant enrichment analysis.


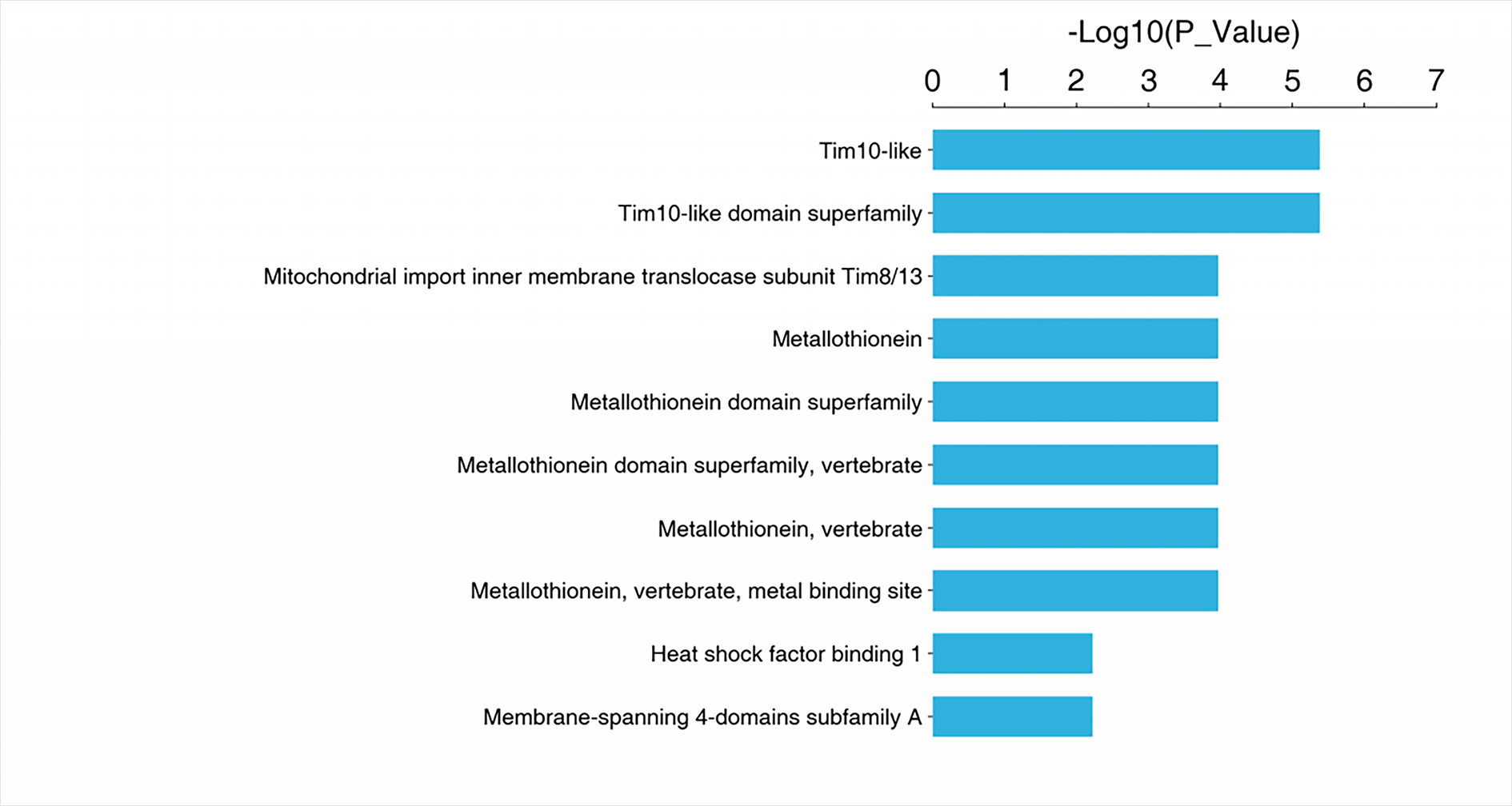


**Supplementary Data S5:** The domain annotation results of the hederagenin-treated group versus the untreated group.

**Original blot images**


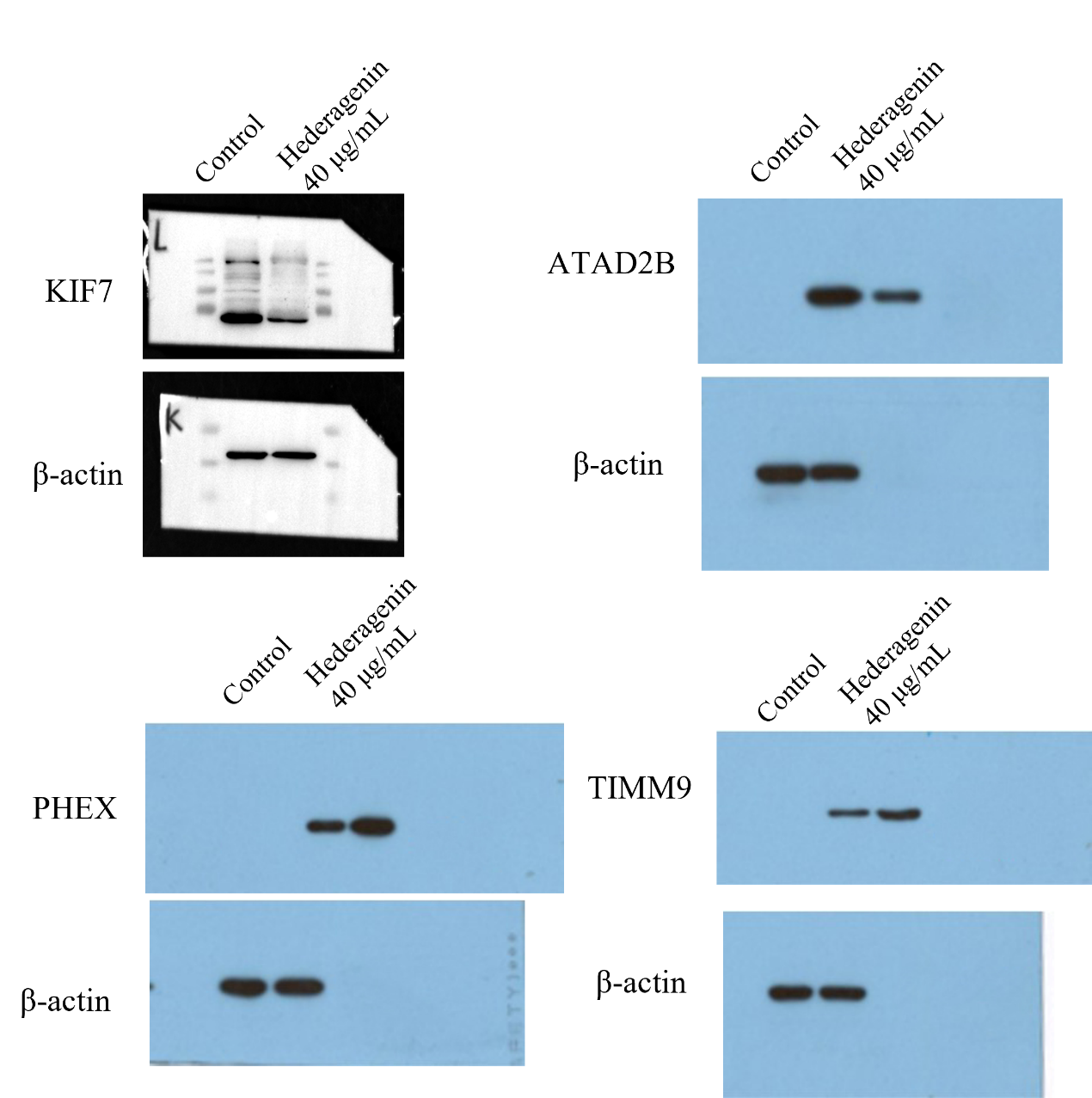

Supplement: Supplementary file 1 — Additional file 1: Supplementary Data S1. SDS-PAGE electrophoresis. Supplementary Data S2. Heat maps of differential protein expression. Supplementary Data S3. The subcellular localization of the differential protein. Supplementary Data S4. Significant enrichment analysis. Supplementary Data S5. The domain annotation results of the hederagenin-treated group versus the untreated group. [file 12953_2023_208_MOESM1_ESM.docx]
